# Supplementary figures and images for: Value of sample information in dynamic, structurally uncertain resource systems
Source: PLoS One. 2018 Jun 29;13(6):e0199326. doi: 10.1371/journal.pone.0199326 (PMC6025880; doi:10.1371/journal.pone.0199326)

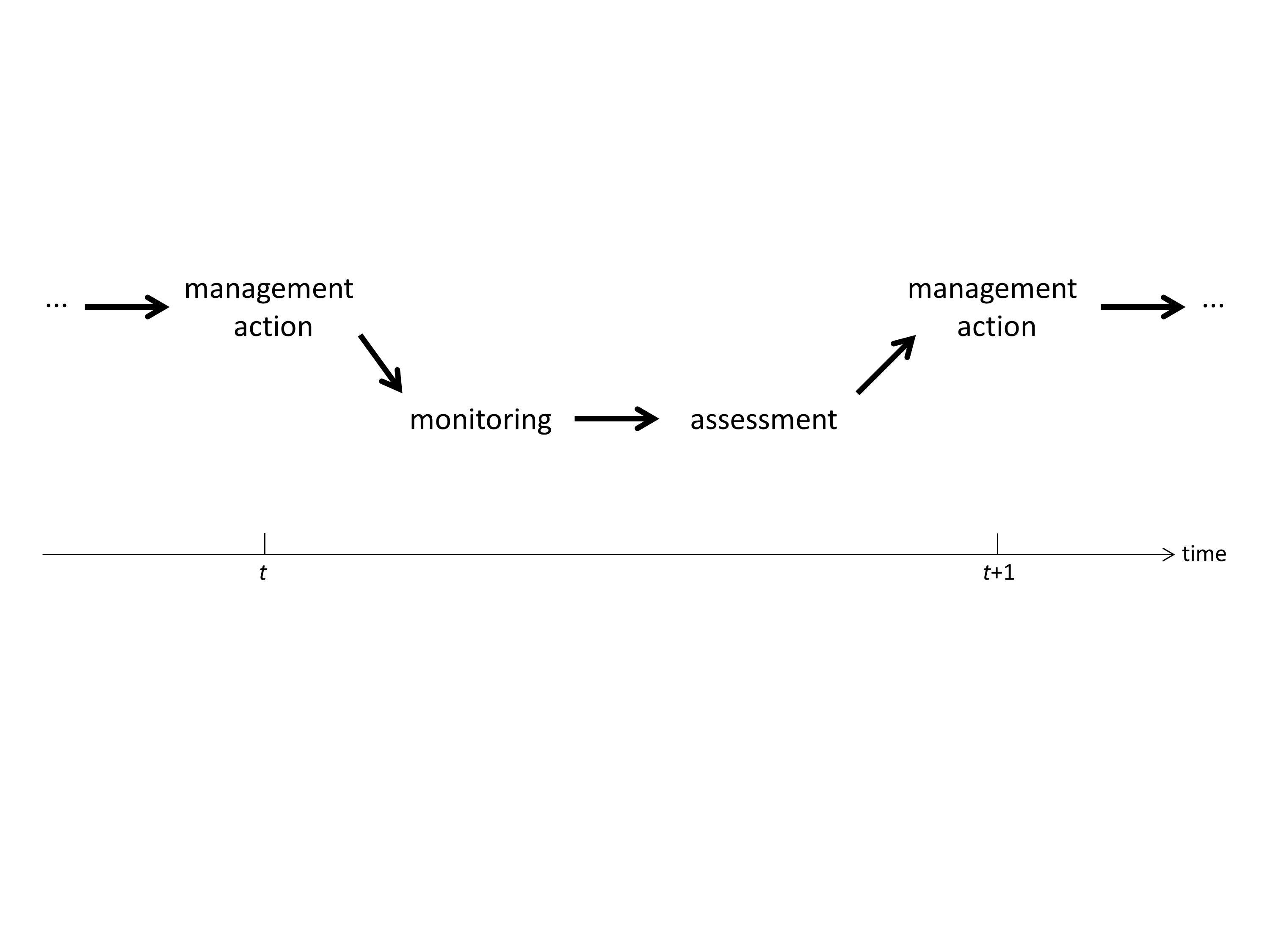

Supplement: S1 Fig — Model 0 assumes routine and intensive burns are equally effective in setting back succession. Model 1 assume intensive burn is more effective. (TIF) [file pone.0199326.s001.tif]
