# Supplementary material for: Value of sample information in dynamic, structurally uncertain resource systems
Source: PLoS One. 2018 Jun 29;13(6):e0199326. doi: 10.1371/journal.pone.0199326 (PMC6025880; doi:10.1371/journal.pone.0199326)
Supplement: S1 File — (DOCX) [file pone.0199326.s002.docx]

**Transition probabilities of Florida scrub and comparison of annual and biennial monitoring**

Transition probabilities of Florida scrub were based on visual classification of low-level, color, high-resolution aerial photography at Merritt Island National Wildlife Refuge. Identification of scrub class was based on a regular grid of square 10-ha cells (Breininger et al. 2010). Each of the available 596 10-ha cells was classified for each of the years 2004-2009 (D. Breininger, Innovative Health Applications, LCC, unpublished data). Transition probabilities were estimated using the maximum-likelihood estimators for a multinomial distribution:

$$\hat{\psi}_{ij}=\frac{n_{ij}}{\sum n_{i}} ,$$

$$\hat{\sigma}\left( \hat{\psi}_{ij} \right)=\sqrt{{\hat{\psi}_{ij}\left( 1-\hat{\psi}_{ij} \right)}/{\sum n_{i}}} ,$$

where $n_{ij}$ is the number of cells that begin in state $i$ and transition to state $j$. Transitions for intensive burns assumed that the manager is able to get a management unit to burn thoroughly. This rarely occurs with routine prescribed burns. Transitions for short-open and short-closed were combined for the intensive burn because of an insufficient sample ($n=9$) of short-open cells that actually carried a fire.

Estimated transition probabilities for each action are:

(a) do nothing:

$$\underline{\hat{\psi}}=\left[ \begin{matrix} & {SO}_{t} & {SC}_{t} & {OO}_{t} & {OC}_{t} & {TM}_{t} \\ {SO}_{t+1} & 0.276 & 0.000 & 0.000 & 0.000 & 0.000 \\ {SC}_{t+1} & 0.310 & 0.484 & 0.028 & 0.011 & 0.003 \\ {OO}_{t+1} & 0.146 & 0.043 & 0.448 & 0.000 & 0.002 \\ {OC}_{t+1} & 0.259 & 0.473 & 0.476 & 0.822 & 0.002 \\ {TM}_{t+1} & 0.009 & 0.000 & 0.048 & 0.167 & 0.993 \end{matrix} \right]$$

(b) routine prescribed burn:

$$\underline{\hat{\psi}}=\left[ \begin{matrix} & {SO}_{t} & {SC}_{t} & {OO}_{t} & {OC}_{t} & {TM}_{t} \\ {SO}_{t+1} & 0.346 & 0.203 & 0.156 & 0.150 & 0.045 \\ {SC}_{t+1} & 0.346 & 0.322 & 0.000 & 0.010 & 0.002 \\ {OO}_{t+1} & 0.192 & 0.051 & 0.558 & 0.163 & 0.012 \\ {OC}_{t+1} & 0.116 & 0.407 & 0.169 & 0.461 & 0.006 \\ {TM}_{t+1} & 0.000 & 0.017 & 0.117 & 0.216 & 0.935 \end{matrix} \right]$$

(c) intensive burn:

$\underline{\hat{\psi}}=\left[ \begin{matrix} & {SO}_{t} & {SC}_{t} & {OO}_{t} & {OC}_{t} & {TM}_{t} \\ {SO}_{t+1} & 0.438 & 0.438 & 0.273 & 0.272 & 0.098 \\ {SC}_{t+1} & 0.156 & 0.156 & 0.000 & 0.006 & 0.005 \\ {OO}_{t+1} & 0.187 & 0.187 & 0.568 & 0.296 & 0.025 \\ {OC}_{t+1} & 0.219 & 0.219 & 0.091 & 0.260 & 0.010 \\ {TM}_{t+1} & 0.000 & 0.000 & 0.068 & 0.166 & 0.862 \end{matrix} \right]$,

where $SO$ = short-open, $SC$ = short-closed, $OO$ = optimal-open, $OC$ = optimal-closed, and TM = tall-max.

Demographic performance for each scrub class is characterized by the expected number of yearlings produced per breeding pair minus the number of breeders per pair that die during the annual cycle. Relative to the do-nothing alternative (action 1), routine and intensive burns (actions 2 and 3, respectively) were arbitrarily assigned a 10% and 40% reduction in demographic performance, respectively, to account for the costs of burning. State and action-specific returns are thus:

$$\left[ \begin{matrix} & 1 & 2 & 3 \\ SO & -0.310 & -0.341 & -0.434 \\ SC & -0.310 & -0.341 & -0.434 \\ OO & 0.490 & 0.441 & 0.294 \\ OC & 0.150 & 0.135 & 0.090 \\ TM & -0.240 & -0.264 & -0.336 \end{matrix} \right]$$

All returns were subsequently normalized to the interval $\left[ 0,1 \right]$ for computational purposes.

We relied on the open-source program MDPSOLVE^©^ (P. Fackler, North Carolina State University), which is a toolbox for the proprietary software MATLAB^©^, to compute the actively adaptive solution to our Markov decision problem. We assumed a finite time horizon ($T=2000$) with no discounting (i.e., $\lambda=1.0$). We used backwards iteration to compute an actively adaptive solution for increments of the prior probability of the null model equal to 0.002.

In the article we demonstrate that *EVSI* can be negative for some system and model states when comparing annual and biennial monitoring schemes. Although this may seem counterintuitive, it follows logically from the mathematical construction provided by Equations 13 and 14. When simulating the optimal policies for annual and biennial monitoring, however, we expect that the annual monitoring scheme should outperform the biennial monitoring scheme. This is in fact the case, as can be seen from their respective long-term cumulative returns for the two alternative models of scrub dynamics; i.e., $q_{t}=1$ and $q_{t}=0$, respectively (Fig S1). Unlike the analytical calculations of state-specific *EVSI* values, the simulations account for the frequency with which various scrub states are visited.

**Literature Cited**

Breininger, D.R., J.D. Nichols, B.W. Duncan, E.D. Stolen, G.M. Carter, D.K. Hunt, and J.H. Drese. 2010. Multistate modeling of habitat dynamics: factors affecting Florida scrub transition probabilities. Ecology 91:3354-3364.
