# Supplementary material for: Correction: Value of sample information in dynamic, structurally uncertain resource systems
Source: PLoS One. 2019 Feb 28;14(2):e0213332. doi: 10.1371/journal.pone.0213332 (PMC6394911; doi:10.1371/journal.pone.0213332)
Supplement: S2 File — (PDF) [file pone.0213332.s002.pdf]

RESEARCH ARTICLE

Value of sample information in dynamic, structurally uncertain resource systems

Byron K. Williams[1☯*], **Fred A. Johnson**[2☯]

1 Renewable Resources Associates, Oakton, VA, United States of America, **2** Wetland and Aquatic Research Center, U.S. Geological Survey, Gainesville, FL, United States of America

☯ These authors contributed equally to this work.
* bkenw111@yahoo.com

Check for updates

OPEN ACCESS

Citation: Williams BK, Johnson FA (2018) Value of sample information in dynamic, structurally uncertain resource systems. PLoS ONE 13(6): e0199326. https://doi.org/10.1371/journal.pone.0199326

Editor: Rob Slotow, University of Kwazulu-Natal, SOUTH AFRICA

Received: January 3, 2018

Accepted: June 5, 2018

Published: June 29, 2018

Copyright: This is an open access article, free of all copyright, and may be freely reproduced, distributed, transmitted, modified, built upon, or otherwise used by anyone for any lawful purpose. The work is made available under the Creative Commons CC0 public domain dedication.

Data Availability Statement: All relevant data are within the paper and its Supporting Information files.

Funding: Funding for this research was provided by the U.S. Geological Survey. Renewable Resources Associates provided support in the form of salaries for authors (BKW), but did not have any additional role in the study design, data collection and analysis, decision to publish, or preparation of the manuscript. The specific roles of these authors are articulated in the "author contribution" section.

Abstract

Few if any natural resource systems are completely understood and fully observed. Instead, there almost always is uncertainty about the way a system works and its status at any given time, which can limit effective management. A natural approach to uncertainty is to allocate time and effort to the collection of additional data, on the reasonable assumption that more information will facilitate better understanding and lead to better management. But the collection of more data, either through observation or investigation, requires time and effort that often can be put to other conservation activities. An important question is whether the use of limited resources to improve understanding is justified by the resulting potential for improved management. In this paper we address directly a change in value from new information collected through investigation. We frame the value of information in terms of learning through the management process itself, as well as learning through investigations that are external to the management process but add to our base of understanding. We provide a conceptual framework and metrics for this issue, and illustrate them with examples involving Florida scrub-jays (*Aphelocoma coerulescens*).

Introduction

Few if any natural resource systems are completely understood and fully observed. Instead, an almost universal situation is for there to be uncertainty about the way a system works and its status at any given time, which can limit effective management (Williams and Johnson [1]). A natural approach to uncertainty is to allocate time and effort to the collection of data, on the assumption that more information will facilitate better understanding and lead to better management. But the collection of more data, either through observation or investigation, requires time and effort that could be put to other activities like conservation on the ground. An important question is whether the use of limited resources to improve understanding is justified by the potential to improve management (Doremus [2]). This question is often asked by managers but only infrequently if ever answered satisfactorily, though some authors (see, e.g., -MaAllister and Pikitch [3] and McAllister and Kirkwood [4]), have used expected resource valuations to contrast different monitoring strategies.

Competing interests: Author (BKW) is proprietor of Renewable Resources Associates. This does not alter the authors' adherence to PLOS ONE policies on sharing data and materials.

There is by now a well-developed theory and approach for the assessment of the value of information in decision making. Raiffa and Schlaifer [5] provided one of the first seminal treatments for the value of information (VOI), coining the name and developing many of its key expressions. Since then many publications have offered descriptions of the value of information, (e.g., Quirk [6], Dakins et al. [7], Yakota and Thompson [8–9], Canessa et al. [10], Williams and Johnson [11]). Keisler et al. [12] provide a comprehensive review of applications of value of information analyses. Several metrics for the value of information are recognized (Yakota and Thompson [8]):

- The expected value of perfect information utilizes an average of optimal model-specific values, averaged over the model likelihoods. The metric consists of this average net of the optimal value in the presence of process uncertainty.
- The expected value of partial information concerns the value added by eliminating uncertainty from a single source, assuming there is more than one source of uncertainty.
- Finally, the expected value of sample information expresses the potential gain in value from the collection of less than perfect information, using a comparison of optimal valuation with additional information against valuation in its absence. A general framework for the value of information that includes perfect, partial and sample information in sequential decision making for natural resources is described by Williams et al. [13].

The expected value of perfect information has been used in a growing number of applications in natural resource management (e.g., Conroy at al. [14], Mäntyniemi et al. [15], Williams et al. [13]), and several applications address the expected value of partial information (e.g., Moore and Runge [16], Johnson et al. [17], Maxwell et al. [18], and Johnson et al. [19]). On the other hand, the number of examples addressing the expected value of sample information is more restricted (e.g., Runge et al. [20], Moore et al. [21], Grantham et al. [22]). Few VOI applications in natural resources deal with dynamic resource systems, in which actions are dependent both on the state of the system and the degree of uncertainty in system dynamics (e.g., Shea et al. [23], Williams and Johnson [11,24], and Moore et al. [21]). Somewhat surprisingly, there are almost no examples for dynamic systems that address the expected value of sample information, even though many resource problems are fundamentally dynamic and a typical monitoring situation involves production of less than perfect information.

In this article we address a change in value from sample information collected during the investigation in dynamic decision making. We frame the value of information in terms of learning through the management process itself, as well as learning through investigations that are external to management but add to our base of understanding. Our objective is to extend valuation to include dynamic decision making with sources of data that are both internal and external to the management process. The framework developed here goes beyond current treatments of the value of sample information in the literature, in its emphasis on management and learning about dynamic natural resources.

In what follows the value of information is described in a context of sequential decision making under uncertainty, with future resource conditions and future understanding potentially influenced by current decisions. We focus specifically on structural uncertainty, that is, uncertainty about the processes that control resource dynamics. Partial observability (Williams [25]), another recognized and important source of uncertainty, can also be addressed by considering additional resources to improve estimates of resource status. However, we emphasize structural uncertainty in this paper, and point the reader to expositions in the literature on valuation under partial observability (Fackler [26], Williams and Johnson [24] and references therein). We provide two examples of the value of sample information based on the management of habitat for the Florida scrub-jay (*Aphelocoma coerulescens*).

Decisions, returns, and uncertainty

Among other things the value associated with sequential decision making under process or structural uncertainty depends on the amount of that uncertainty. With greater understanding one can make more informed (and higher valued) decisions; with less understanding progress toward achieving resource goals and objectives is limited.

Here we assume a managed natural resource (e.g., a landscape, an amphibian population, a butterfly colony, the number of vegetative organism in an area) that is subject to only partial understanding. Uncertainty about how the resource system works is expressed by means of different hypotheses (models) about the system and its responses to management actions. Each model has a measurable likelihood of being the most appropriate, based on current information and understanding (Williams et al. [27]).

We also assume a range of different management actions (e.g., different seeding mixes, harvest strategies, water control regimes, geographic locations), with time-specific actions influencing the transition of the resource from its current state to a future state, and generating returns that provide a basis for comparing different management actions. Once an action is taken and a transition is made to a new state, another action is taken, and another return is generated at that time. The trajectory of anticipated returns depends on which hypothesis (model) is most appropriate, and therefore inherits the model uncertainty.

The challenge in such a situation is to recognize and measure the change in value resulting from an increase in information and understanding. A broadly accepted measure of change is given by a comparison of optimal valuation produced with additional information, against optimal valuation in its absence (Raiffa and Schlaifer [5]). An understanding of the change in value enables assessment of cost-effectiveness in targeting uncertainty with additional research or monitoring.

Decision making under structural uncertainty

A framework for the expected value of sample information under dynamic decision making applies to resources that are subject to management through time. Both resource status and management interventions are seen as fluctuating through time, with the system state and action at time t influencing system behavior going forward. Here we summarize the components of learning-based management under structural uncertainty. The necessary notation is highlighted in Table (1).

System dynamics. State transitions are described in terms of Markov decision processes (MDP) (Puterman [28], Williams et al. [27]): If x_t and a_t are the state and action at a particular

Table 1. Notation used to characterize dynamic decision making and valuation under structural uncertainty.

t	Time index for a range of times constituting the time frame. The index is assumed here to take positive integer values, from some time t_0 through time T that may be infinite.
x_t	System state (e.g., size, density, spatial coverage). Because the system is assumed to change through time its state is time-specific.
k	Model index for $k = 1,\ldots,K$ models representing different hypotheses about system dynamics.
$\underline{q}_t$	Vector $(q_t(1),q_t(2),\ldots,q_t(K))$ of model-specific probabilities, with $q_t(k)$ the probability that model k best represents the system at time t.
a_t	Action taken as a result of decision making. Because they are taken through time, actions are time-indexed.
A_t	Policy that specifies a particular action for each system state and model state at each time starting at time t in the time frame.
$R(a_t, x_t)$	Return corresponding to action a_t and system state x_t.

https://doi.org/10.1371/journal.pone.0199326.t001

time t and x_{t+1} is the state at the next time, then the probability of transition from x_t to x_{t+1} is $P(x_{t+1} \mid x_t, a_t)$.

Under structural uncertainty the decision process is not completely understood, i.e., the transition probabilities in $P(x_{t+1} \mid x_t, a_t)$ are uncertain (Williams [29], Williams and Brown [30]). Different Markovian models $P_k(x_{t+1} \mid x_t, a_t)$ are used along with model probabilities $q_t(k)$ to account for structural uncertainty. The model state $\underline{q}_t = (q_t(1), q_t(2), \ldots, q_t(K))$ evolves through time as information accumulates via monitoring, and an average of model-specific transition probabilities based on $\underline{q}_t$ produces model-averaged transition probabilities

$$\bar{P}(x_{t+1}|x_t, a_t, \underline{q}_t) = \sum\nolimits_k q_t(k) P_k(x_{t+1}|x_t, a_t).$$

Decision making. A policy A_t of actions over time frame $\{t,\ldots,T\}$ consists of actions $A(x_t, \underline{q}_t)$ for each system and model state at each time t in the time frame. Policy A_t can be characterized sequentially by action a_t at time t, followed thereafter by the remainder A_{t+1} of the policy over $\{t + 1,\ldots,T\}$:

$$A_t = \{A(x_t, \underline{q}_t), A_{t+1}\} = \{a_t, A_{t+1}\}.$$

Propagating uncertainty. The dynamics of the model state are driven by information produced over time that is either internal or external to management. The source of information for internal updating comes from within the management process itself, in the spirit of adaptive management (Nichols and Williams [31]). Bayes' theorem (Lee [32]) can be used for updating uncertainty, based on system state transitions from x_t to x_{t+1}:

$$q_{t+1}(k) = \frac{q_t(k) P_k(x_{t+1}|x_t, a_t)}{\bar{P}(x_{t+1}|x_t, a_t, \underline{q}_t)}. \quad (1)$$

Uncertainty also can be updated with information from outside the management process, that is, from experimentation or tracking that is effectively independent of decision making (Williams [33]). In this case resource data z_t are acquired through external investigation, with Bayes' theorem again used for updating uncertainty based on model-specific data distributions:

$$\begin{aligned} q'_t(k) &= \frac{q_t(k) P_k(z_t)}{\sum_k q_t(k) P_k(z_t)} \\ &= \frac{q_t(k) P_k(z_t)}{\bar{P}(z_t|\underline{q}_t)}. \end{aligned} \quad (2)$$

Uncertainty updating with both sources of information factors into the expected value of sample information with dynamic decision making.

Valuation. Strategy valuation for this problem is based on the accrual of returns $R(a_t, x_t)$ through time, with each return incorporating the costs and benefits corresponding to action a_t when the system is in state x_t. A value function $V(A_t|x_t, \underline{q}_t)$ for decision making aggregates returns starting at time t:

$$V(A_t|x_t, \underline{q}_t) = E\left[\sum\nolimits_{\tau=t}^{T} R(a_\tau, x_\tau)|x_t, \underline{q}_t\right], \quad (3)$$

where the expectation accounts for stochastic transitions among states as well as the structural uncertainty represented by multiple models and their likelihoods. Step-wise updating of the

value function is given by

$$V(A_t|x_t, \underline{q}_t) = R(a_t, x_t) + \sum_{x_{t+1}} \bar{P}(x_{t+1}|x_t, a_t, \underline{q}_t) V(A_{t+1}|x_{t+1}, \underline{q}_{t+1}). \quad (4)$$

The expression $V(A_t|x_t, \underline{q}_t)$ serves as a value or objective function by which to compare and contrast the effectiveness of different management strategies.

Learning-based management. Decision making with internal learning as described above characterizes an adaptive approach to management (Williams [29]), whereby adjustments to decision making occur as understanding improves with the ultimate goal of improved management (Walters [34]). Adaptive management is promoted through a sequence of (*i*) decision making and taking actions, (*ii*) followed by monitoring of system responses, (*iii*) followed by assessment of data, (*iv*) followed by the integration of what is learned into future decision making (**Fig 1**).

Adaptive management can be either active or passive, with active adaptive management incorporating the potential for learning directly into the process of decision making (Williams [35]). Optimal decision making is given by

$$V[x_t, \underline{q}_t] = \max_{a_t} \left\{ R(a_t, x_t) + \lambda \sum_{x_{t+1}} \bar{P}(x_{t+1}|x_t, a_t, \underline{q}_t) V[x_{t+1}, \underline{q}_{t+1}] \right\}, \quad (5)$$

where the updated model state $\underline{q}_{t+1}$ in $V[x_{t+1}, \underline{q}_{t+1}]$ indicates the use of learning in identification of strategy (see Appendix).

On the other hand, passive adaptive management can be described in terms of the absence of an explicit accounting of learning in the choice of strategy (Williams [35]):

$$V[x_t, \underline{q}_t] = \max_{a_t} \{ R(a_t, x_t) + \lambda \sum_{x_{t+1}} \bar{P}(x_{t+1}|x_t, a_t, \underline{q}_t) V[x_{t+1}, \underline{q}_t] \}, \quad (6)$$

where the prior model state $\underline{q}_t$ in $V[x_{t+1}, \underline{q}_t]$ indicates an absence of learning in the identification of decisions (see Appendix). In the development below, the expected value of sample information is described in terms of both active and passive adaptive management.

Combining internal and external learning in *EVSI*

Under sequential decision making, an approach to the expected value of sample information is to include information internal to the management process as above, along with experimentally generated information from outside the management process. The value obtained can

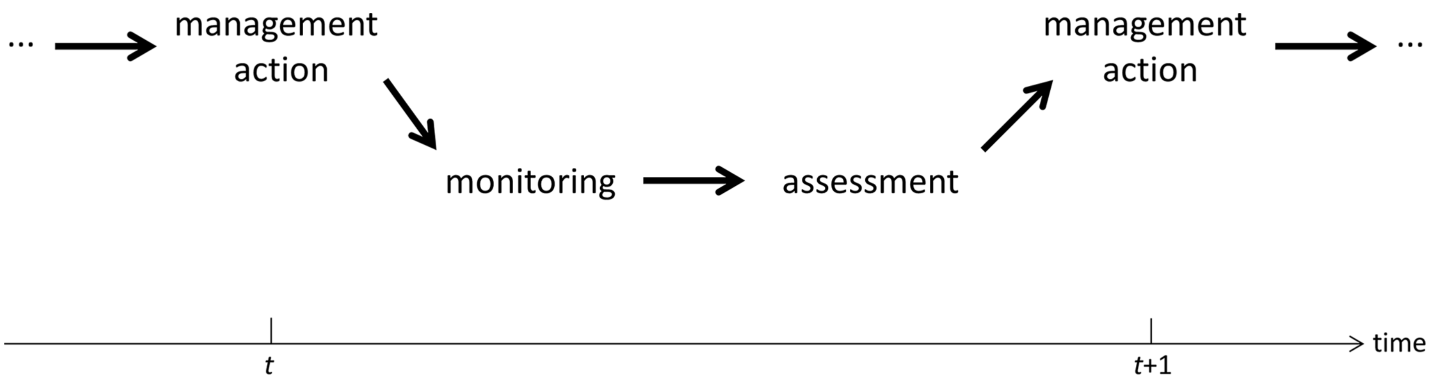

Fig 1. Adaptive management, with a repeated sequencing through time of decision making and taking actions; followed by monitoring of system responses; followed by assessment of data; followed by the integration of what is learned into future decision making.

https://doi.org/10.1371/journal.pone.0199326.g001

then be compared with the value produced with internal learning only, to assess the net benefit of the experimentation.

The learning process in this situation involves updating the prior model state $\underline{q}_t$ to $\underline{q}_t'$ with external information as in Eq (2), and then using the update in iterative valuation as in Eq (5). In combination, external and internal learning can accelerate the rate of learning, by allowing the model state to be updated prior to its use in optimal valuation. Preposterior updating (Berger [36]) with probabilities for the data z_t is given by

$$\sum_{z_t} \bar{P}(z_t|\underline{q}_t) V[x_t, \underline{q}_t'] = \sum_{z_t} \bar{P}(z_t|\underline{q}_t) \max_{a_t} \left\{ R(a_t, x_t) + \sum_{x_{t+1}'} \bar{P}(x_{t+1}|x_t, a_t, \underline{q}_t') V[x_{t+1}, \underline{q}_{t+1}] \right\} \quad (7)$$

(see Appendix), with the posterior model state $\underline{q}_{t+1}$ based on the prior model state q_t'. Preposterior updating provides a measurement of value before data z_t are known and actions are taken. The expected value of sample information is then expressed as the difference

$$\sum_{z_t} \bar{P}(z_t|\underline{q}_t) V[x_t, \underline{q}_t'] - V[x_t, \underline{q}_t], \quad (8)$$

where $\underline{q}_t$ is updated to $\underline{q}_t'$ based on data z_t with distribution $\bar{P}(z_t|\underline{q}_t) = \sum_k q_t(k) P_k(z_t)$ (see Eq (2)). The first term in Eq (8) is an average optimal valuation from Eq (7) resulting from the updating of the model state with external data. The second term is an optimal valuation based on the current system and model state. The difference expresses the marginal value expected with new sample information. *EVSI* can be seen to be state-dependent, in that the value given by the comparison in Eq (8) is conditional on the particular combination $(x_t, \underline{q}_t)$ of system and model states. That is, different combinations of system and model states can produce different values.

The use of passive adaptive management in *EVSI* proceeds in much the same way, except that the updating of model state in the decision making process involves the use of $\underline{q}_t'$ rather than $\underline{q}_{t+1}$ in the valuation:

$$\sum_{z_t} \bar{P}(z_t|\underline{q}_t) V[x_t, \underline{q}_t'] = \sum_{z'} \bar{P}(z_t|\underline{q}_t) \max_{a_t} \left\{ R(a_t, x_t) + \sum_{x_{t+1}} \bar{P}(x_{t+1}|x_t, a_t, \underline{q}_t') V[x_t, \underline{q}_t'] \right\}. \quad (9)$$

As above, the difference between active and passive adaptive management is the incorporation of anticipated learning in active adaptive management, as reflected in the updated model state $\underline{q}_{t+1}$ in the value term $V[x_{t+1}, \underline{q}_{t+1}]$ in Eq (7).

A simple illustration of the use of internal and external information involves the adaptive management on provincial lands of a particular ecological type, and an investigation under fixed management is also being conducted on a nearby federal conservation area of the same type. Assuming that monitoring and model state updating with Eq (2) occur somewhat earlier on the federal lands, information from the updating can be made available to inform decision making on the provincial lands. If the resource situation at the 2 locations is similar in the biological structures and environmental drivers, then folding what is learned on the federal lands into learning-based decision making on the provincial lands (Eqs (5) and (6)) should increase the rate of learning on the provincial lands, and lead to a more rapid improvement in their management. *EVSI* at any point in the decision process is simply the comparison of an average valuation $\sum_{z_t} \bar{P}(z_t|\underline{q}_t) V[x_t, \underline{q}_t']$ that accounts for new information from the federal lands, against the valuation $V[x_t, \underline{q}_t]$ in the absence of any new information from that source. Using *EVSI* allows one to recognize the potential for additional value to provincial land management by monitoring on the federal lands.

Example: Habitat management for the Florida scrub-jay

The Florida scrub-jay is an endemic species that is designated as threatened under the Endangered Species Act (Root [37], Stith et al. [38]). Scrub-jays are restricted to Florida scrub (hereafter, "scrub"), which is a rare habitat characterized by evergreen, xeromorphic shrubs including oaks, repent palms (*Serenoa repens*, *Sabal etonia*), and ericaceous shrubs (*Lyonia* spp., *Vaccinium* spp.) (Foster and Schmalzer [39]). Scrub is maintained by frequent fire, and landscape fragmentation and fire suppression have resulted in many scrub communities that are no longer capable of supporting scrub-jay populations (Breininger and Carter [40]). Prescribed burning has thus become the primary management tool in reserves where the viability of scrub-jays and other scrub species is an important objective.

Of the many scrub attributes affecting scrub-jay demography (Breininger et al. [41]), perhaps the most important is scrub height (Breininger et al. [42], Breininger and Carter [40]). Scrub height is classified as short (<120 cm), optimal (120–170 cm), or tall-mix (>170 cm) (Breininger and Carter [40]). Short and optimal height scrub are further classified as open (>50% of the scrub containing bare ground) or closed. Optimal-height scrub acts as a reliable source habitat for jays, whereas the other classes always act as demographic sinks (Breininger and Oddy [43]). The goal of a manager is to maximize the cumulative demographic performance of scrub-jays over time, net the cost of conducting prescribed burns.

For the purposes of this example, we assume a management unit that is homogenous, with one-year transition probabilities for each scrub class along with do-nothing and prescribed-burn management actions (**S1 File**). We also allow for an intensive burn to ensure that the entire management unit is effectively burned. Our null model posits that routine and intensive burns are equally effective (or ineffective) at setting back succession, though an intensive burn is more expensive due to the need to guard against greater threats to infrastructure and public safety. Thus, an intensive burn is never optimal under the null model. The alternative model posits that intensive burns are more effective at setting back succession than routine burns, and thus would be used when their greater short-term cost is offset by greater demographic performance of scrub-jays over the long term. The optimal, actively adaptive policy is depicted in **Fig 2**, in which the optimal management action is a function of both scrub state (i.e., system state) and the probability of the null model (i.e., model state). The optimal action can be an intensive burn as long as there is at least some probability (≥ 0.002) of the alternative model being correct. But even in those cases, an intensive (and more expensive) burn is only optimal for the most fire-resistant states (short-closed, optimal-closed, and tall-mix). State and action-specific transition probabilities and returns, and computational details for the actively adaptive policy are provided in Supporting Information (**S1 File**).

In this example we assume that data external to the management process are available, and we wish to know the contribution of the external data for improving the management process. Suppose a researcher has the ability to observe the effect of an intensive burn at another site prior to decision making for the management unit in question. We first calculated the Expected Value of Perfect Information (*EVPI*; Johnson and Williams [44]) (see Appendix), and then calculated *EVSI* for each combination of scrub state and probability of the null mode according to Eq (9). Some authors (e.g., Walters [34], Moore and McCarthy [45]) have observed that *EVPI* is often low in practice, which is the case in our scrub-jay example (**Fig 3**). Expressed as a percentage gain in expected objective value, the value of eliminating model uncertainty is always < 1%. This can actually be good news for a manager, in that there is little incentive to eliminate model uncertainty; a management policy based on an average model may be sufficient. As expected, values of *EVPI* are considerably higher than those of *EVSI*, and are at a maximum in the interior of the model state. *EVPI* is uniformly higher for tall-mix,

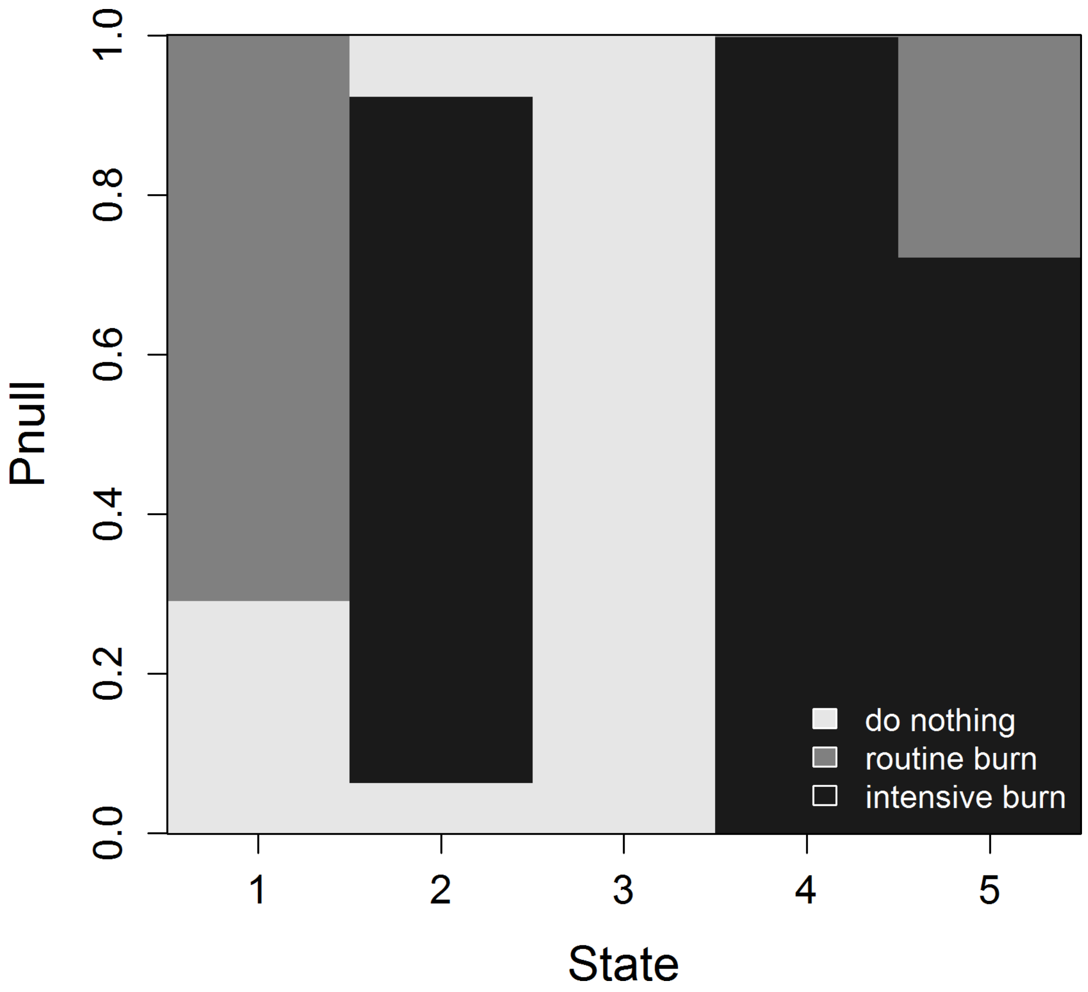

Fig 2. The optimal, actively adaptive management policy to maximize demographic performance of Florida scrub-jays. Scrub states are: (1) short-open; (2) short-closed; (3) optimal-open; (4) optimal-closed; and (5) tall-mix. Pnull is the probability of the null model, which posits that an intensive burn is no more effective at restoring optimal height scrub than a routine burn. An intensive burn can be optimal for short-closed, optimal-closed, and tall mix scrub states, but only if the alternative model, which assumes an intensive burn is more effective than a routine burn, has a probability ≥ 0.002 (i.e, Pnull = 1–0.002 = 0.998, or near certainty about the null model).

https://doi.org/10.1371/journal.pone.0199326.g002

which is the scrub state most resistant to fire. In contrast, *EVSI* is uniformly higher for short-closed, suggesting that experimenting with intensive burns in this scrub state would provide the greatest short-term gain in management performance. However, the advantage of observing a single intensive burn that is external to the management process provides little advantage because both the null and alternative models have broad overlap in their transition probabilities (see Supporting Information), and thus model discrimination is very difficult.

Constraints on the sequencing of monitoring

A somewhat different approach to *EVSI* with sequential decision making involves monitoring that can be less frequent than decision making. Consider resource management in which actions are chosen annually, whereas monitoring can be conducted either biennially or annually. Under these conditions one can meaningfully assess the value of the additional information produced by annual rather than biennial monitoring. The question is how much value would be added.

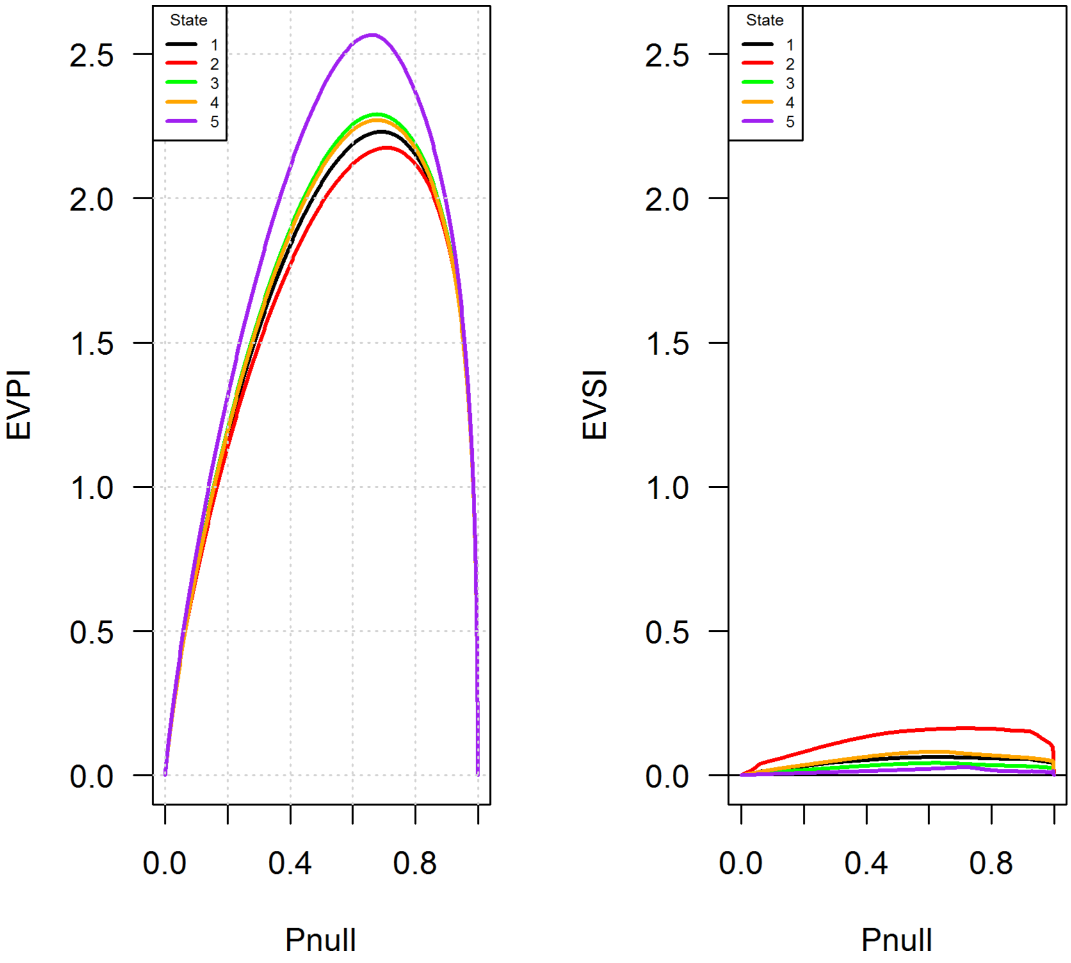

Fig 3. Left panel: The Expected Value of Perfect Information (*EVPI*) for eliminating uncertainty about the most appropriate model governing the effects of fire on habitat for Florida scrub-jays. Right panel: The Expected Value of Sample Information (*EVSI*) resulting from the use of an experimental, intensive burn. Scrub states are: (1) short-open; (2) short-closed; (3) optimal-open; (4) optimal-closed; and (5) tall-mix. Pnull is the probability of the null model, which posits that an intensive burn is no more effective at restoring optimal height scrub than a routine burn.

https://doi.org/10.1371/journal.pone.0199326.g003

To determine the value produced by the additional monitoring, we compare valuation with annual versus biennial monitoring. In any year t, valuation for annual monitoring is given by Eq (4),

$$V(A_t|x_t, \underline{q}_t) = R(a_t, x_t) + \sum_{x_{t+1}} \bar{P}(x_{t+1}|x_t, a_t, \underline{q}_t) V(A_{t+1}|x_{t+1}, \underline{q}_{t+1}),$$

with optimal valuation shown in Eq (5):

$$V[x_t, \underline{q}_t] = \max_{a_t} \left\{ R(a_t, x_t) + \lambda \sum_{x_t} \bar{P}(x_{t+1}|x_t, a_t, \underline{q}_t) V[x_{t+1}, \underline{q}_{t+1}] \right\}.$$

Because system status is observed every year, valuation in successive years t and t+1 have the same form, with the value function for t+1 replicating that for year t simply by incrementing the time index by 1:

$$V(A_{t+1}|x_{t+1}, \underline{q}_{t+1}) = R(a_{t+1}, x_{t+1}) + \lambda \sum_{x_{t+2}} \bar{P}(x_{t+2}|x_{t+1}, a_{t+1}, \underline{q}_{t+1}) V(A_{t+2}|x_{t+2}, \underline{q}_{t+2})$$

and

$$V[x_{t+1}, \underline{q}_{t+1}] = \max_{a_{t+1}} \left\{ R(a_{t+1}, x_{t+1}) + \lambda \sum_{x_{t+1}} \bar{P}(x_{t+2}|x_{t+1}, a_{t+1}, \underline{q}_{t+1}) V[x_{t+2}, \underline{q}_{t+2}] \right\}.$$

The situation is somewhat different for biennial monitoring, where the system state is observed in a given year *t*, not observed in the subsequent year *t*+1, observed again in year *t*+2, and so on. Because the observed states x_t to x_{t+2} can be combined with model-specific transition probabilities to determine model state $\underline{q}_{t+2}$ by Bayes' theorem (Williams and Johnson 2017), one to compute a 2-step value function

$$\begin{aligned} V(A_t|x_t, \underline{q}_t) = R(a_t, x_t) + \lambda \sum_{x_{t+1}} \bar{P}(x_{t+1}|x_t, a_t, \underline{q}_t) \times \\ \left\{ R(a_{t+1}, x_{t+1}) + \lambda \sum_{x_{t+2}} \bar{P}(x_{t+2}|x_{t+1}, a_{t+1}, \underline{q}_{t+1}) V(A_{t+2}|x_{t+2}, \underline{q}_{t+2}) \right\}, \end{aligned} \quad (10)$$

which in turn can be maximized over $A_t = \{a_t, a_{t+1}, A_{t+2}\}$ to produce $V[x_t, \underline{q}_t]$ for each combination $(x_t, \underline{q}_t)$. For a year *t* in which biennial monitoring occurs, the valuation in Eq (10) can be shown to be identical to valuation in Eq (4) for annual monitoring (Williams and Johnson [46]). It follows that there is no difference in value between the monitoring scenarios, i.e., no value is added in switching from biennial to annual monitoring in a year *t* in which biennial monitoring occurs.

On the other hand, for year *t*+1 when biennial monitoring does not occur, there is a difference in the valuations for annual and biennial monitoring, because x_{t+1} and $\underline{q}_{t+1}$ are not identified in the latter scenario. However, x_{t+1} and $\underline{q}_{t+1}$ are related stochastically to x_t and $\underline{q}_t$, which are known through monitoring. Averaging over the transition probabilities $\bar{P}(x_{t+1}|x_t, a_t, \underline{q}_t)$ produces a valuation for year *t*+1,

$$\bar{V}(A_{t+1}|x_t, \underline{q}_t, a_t) = \sum_{x_{t+1}} \bar{P}(x_{t+1}|x_t, a_t, \underline{q}_t) V(A_{t+1}|x_{t+1}, \underline{q}_{t+1}), \quad (11)$$

and using a_t^*, a_{t+1}^* and A_{t+2}^* from the optimization of $V(A_t|x_t, \underline{q}_t)$ in Eq (10) produces the optimal valuation

$$\bar{V}[x_t, \underline{q}_t, a_t^*] = \sum_{x_{t+1}} \bar{P}(x_{t+1}|x_t, a_t^*, \underline{q}_t) V[x_{t+1}, \underline{q}_{t+1}] \quad (12)$$

for year *t*+1 (Williams and Johnson [46]). The change in valuation for the 2 monitoring scenarios is therefore given by a comparison of the valuation $V[x_{t+1}, \underline{q}_{t+1}]$ for annual monitoring, and the average valuation $\bar{V}[x_t, \underline{q}_t, a_t^*]$ for biennial monitoring:

$$V[x_{t+1}, \underline{q}_{t+1}] - \bar{V}[x_t, \underline{q}_t, a_t^*]. \quad (13)$$

This measure of value, which is directly related to an increase in the frequency of monitoring, can prove useful to managers in determining whether to reduce annual to biennial monitoring, or to expand biennial to annual monitoring.

Example: Habitat monitoring for the Florida scrub-jay

The Florida scrub-jay management problem described above can be used to illustrate the effect of an increased monitoring frequency. We calculated actively adaptive management policies for annual and biennial monitoring schemes (Table 2). The marginal value in Eq (13) varies depending on system and model state; in fact it is negative for some states (McDonald and Smith [47]). Because an average of the optimal values $V[x_{t+1}, \underline{q}_{t+1}]$ is compared against an

Table 2. Optimal actions (a^*) and cumulative values (V) over 2000 time steps for managing habitat for Florida scrub-jays under annual and biennial monitoring schemes. The Expected Value of Sample Information (*EVSI)* is the difference in expected performance between the two monitoring schemes. Scrub states x_t are: (1) short-open; (2) short-closed; (3) optimal-open; (4) optimal-closed; and (5) tall-mix. Model state q_t is the probability of the null model, which posits that an intensive burn is no more effective at restoring optimal height scrub than a routine burn. Optimal actions a^* are: (1) do nothing; (2) routine burn; and (3) intensive burn. Sometimes the biennual-monitoring policy $a^*_{t+1}|x_t, q_t, a^*_t$ has actions that differ from those for the annual-monitoring policy a^*_t because in the t+1 years monitoring information is unavailable in the former policy and actions have to be conditioned on the system state, model state, and action for the previous year t.

Scrub state x_t	Model state q_t	Annual monitoring		Biennial monitoring		*EVSI*
		a^*_t	$V[x_t,q_t]$	$a^*_{t+1}\|x_t, q_t, a^*_t$	$\bar{V}[x_t, q_t, a^*_t]$	
1	0.0	1	763.23	1	768.45	-0.22
1	0.5	2	702.35	3	703.41	-1.06
1	1.0	2	640.56	2	640.87	-0.31
2	0.0	1	768.19	3	768.87	-0.68
2	0.5	3	702.35	3	705.11	-2.77
2	1.0	1	640.46	2	640.94	-0.47
3	0.0	1	769.51	3	768.93	0.58
3	0.5	1	703.53	1	702.82	0.71
3	1.0	1	641.78	2	641.08	0.70
4	0.0	3	768.60	1	768.36	0.24
4	0.5	3	702.57	3	704.53	-1.96
4	1.0	2	640.74	2	640.43	0.31
5	0.0	3	766.48	3	766.74	-0.26
5	0.5	3	700.22	3	702.77	-2.55
5	1.0	2	638.71	2	638.85	-0.13

https://doi.org/10.1371/journal.pone.0199326.t002

optimal value for one particular state x_{t+1} that may be included in that average, Eq (13) may be negative or positive, depending on both the transition probabilities $\bar{P}(x_{t+1}|x_t, a^*_t, \underline{q}_t)$ and the associated optimal valuations in Eq (12). Consider, for example, a system and model state combination $(x_{t+1}, \underline{q}_{t+1})$ that can be reached from $(x_t, \underline{q}_t)$. If $V[x_{t+1}, \underline{q}_{t+1}]$ is large but the corresponding probability $\bar{P}(x_{t+1}|x_t, a_t, \underline{q}_t)$ is small, the comparison in Eq (13) may be positive. On the other hand, a small value $V[x_{t+1}, \underline{q}_{t+1}]$ coupled with a small probability $\bar{P}(x_{t+1}|x_t, a_t, \underline{q}_t)$ may produce a negative value.

More data from annual monitoring should produce increased value on average over the long term, a result borne out from long-term simulations that account for the likelihood of occurrence for different states (**S1 Fig**). Nonetheless, the advantage of annual monitoring over biennial monitoring appears to be very small in this example, probably because of the strong relationship between states in successive years. This confirms the intuitive result that there is little to be gained from the frequent monitoring of slowing changing ecosystems.

Discussion

There is a long record of advances in understanding the processes influencing resource dynamics, in modeling resource behaviors, in the recognition of resource patterns, and in methodologies for resource monitoring and estimation. On the other hand, decision making, including a framework for valuation, continues to lag behind natural resources science, despite the growth in operations research and decision science (Schwartz et al. [48]). A technical framework is needed for the evaluation of costs and consequences of resource decisions, so as to allow a comparative assessment of alternative strategies. With such a framework it then becomes possible to assess the limitations of uncertainty on decision making, and the value of eliminating that uncertainty.

In this paper we offer an assessment framework for strategy valuation that builds on adaptive management and the value of information. The general goal is to facilitate the assessment of monitoring in the decision making process, through the consideration of additional value accruing to additional sampling information. The expected value of sample information serves as a metric by which managers can explicitly compare the benefit of extended data collection against associated opportunity and other costs, thereby facilitating smart decision making based on the efficiency of the additional effort. Advances have been made in recent years in the value of information with one-time decision making. In this paper we expand on that work, to address the relatively common occurrence in natural resources of sequential decision making and monitoring over an extended time frame.

In the above treatment of internal and external monitoring we focused on the marginal value of external data collection, on the assumption that it could supplement an ongoing process of internal monitoring. It should be noted that an analogous assessment is possible, whereby external investigation is ongoing and it is internal monitoring that is considered to be supplemental to it. Framing the issue in this way would allow managers to consider whether to implement (or continue) internal monitoring as part of the management process based on the marginal value of doing so, or to rely on externally collected data only.

As to the cadence of monitoring, we note that it is possible to extend the period between monitoring events so that monitoring occurs less frequently than every other year. Consider the prospect of triennial monitoring, in which a monitoring effort is mounted every 3 years. A computing form for valuation would mirror that shown above, except it would need to account for state transitions over 3 years. Again, the valuations for annual and triennial monitoring would be equivalent for years in which monitoring occurs, but would differ in years when there is no monitoring. However, there would be different valuations for the non-monitoring years, leading to a differential value-added for annual monitoring that would depend on the out-year under consideration.

When using *EVSI* to explore the value of additional information to resolve uncertainty, it is important not to misinterpret results (Johnson et al. [49]). One such misinterpretation is to conclude that a low value of *EVSI* means monitoring is unneeded. As indicated above, *EVSI* is a comparison of an average of optimal values produced with additional sample information, versus the optimal value that is attainable in the absence of additional information (Eq 10). As such it is effectively a marginal analysis, addressing the value of additional monitoring that contributes to an ongoing if imperfect monitoring effort that informs decision making. Monitoring is required for the state-based information on which the optimal resource decision making depends, and the question here is whether additional monitoring is justified by the potential increase in value that would be produced. A decision to increase or decrease the monitoring effort relies on the answer to this question. Whether to terminate monitoring altogether is a quite different question, one that is not addressed by examining the effect of a marginal change in monitoring effort (Williams and Johnson [24]).

Finally, we emphasize that as potentially useful as the value of information is, and in particular *EVSI*, these metrics only partially characterize the benefit to be derived from the decision framework presented above. Management objectives, potential actions, sources of uncertainty, and forecasts of resource responses provide a decision making "architecture" for post-decision monitoring and assessment that can track resource responses and evaluate progress toward objectives. A technical assessment of the value of the information produced can certainly contribute in informing management. However, the metrics are certainly not the only, and possibly not even the most relevant, measures of value for the decision framework. Among other things, a systematic and structured accounting of the elements of decision making can facilitate collaboration and shared decision making, lowering the potential for contentiousness and conflict among stakeholders

(Nichols et. al [50]). The value of information can certainly contribute to, but should not obscure, these and other benefits accruing to a structured process of decision making.

Appendix

- We first consider optimal valuation with internal monitoring. Action taken at each time maximizes the sum of current return and expected future value. Two decision making approaches are active adaptive management and passive adaptive management, and strategy valuation applies to both.
  Active adaptive management
  Expected future value is based on updated model state $\underline{q}_{t+1}$:
  $$\begin{aligned} V[x_t, \underline{q}_t] &= \max_{A_t} V(A_t | x_t, \underline{q}_t) \\ &= \max_{\{a_t, A_{t+1}\}} \left\{ R(a_t, x_t) + \lambda \sum_{x_{t+1}} \bar{P}(x_{t+1} | x_t, a_t, \underline{q}_t) V(A_{t+1} | x_{t+1}, \underline{q}_{t+1}) \right\} \\ &= \max_{a_t} \left\{ R(a_t, x_t) + \lambda \sum_{x_{t+1}} \bar{P}(x_{t+1} | x_t, a_t, \underline{q}_t) V[x_{t+1}, \underline{q}_{t+1}] \right\}. \end{aligned}$$
  Passive adaptive management
  Expected future value is based on current model state $\underline{q}_t$:
  $$\begin{aligned} V[x_t, \underline{q}_t] &= \max_{A_t} V(A_t | x_t, \underline{q}_t) \\ &= \max_{\{a_t, A_{t+1}\}} \left\{ R(a_t, x_t) + \lambda \sum_{x_{t+1}} \bar{P}(x_{t+1} | x_t, a_t, \underline{q}_t) V(A_{t+1} | x_{t+1}, \underline{q}_t) \right\} \\ &= \max_{a_t} \left\{ R(a_t, x_t) + \lambda \sum_{x_{t+1}} \bar{P}(x_{t+1} | x_t, a_t, \underline{q}_t) V[x_{t+1}, \underline{q}_t] \right\}. \end{aligned}$$
  The expected value of perfect information (*EVPI*) can be calculated with either approach. *EVPI* compares the average optimal valuation, assuming complete understanding, against optimal valuation under structural uncertainty:
  $$EVPI = \sum_k q_t(k) V_k[x_t] - V[x_t, \underline{q}_t].$$

EVPI is necessarily non-negative (Williams and Johnson 2015*b*).

- Next we consider optimal valuation with internal and external monitoring. Here we utilize preposterior averaging of optimal adaptive valuations:
  Step 1. Update $\underline{q}_t$ to $\underline{q}'_t$ using external data z_t as in Eq (2).
  Step 2. Use $\underline{q}'_t$ in the optimal valuation in Eq (5).
  Step 3. Average the optimal valuations in step 2 over the data z_t that produce $\underline{q}'_t$:
  $$\begin{aligned} \sum_{z_t} \bar{P}(z_t | \underline{q}_t) V[x_t, \underline{q}'_t] &= \sum_{z_t} \bar{P}(z_t | \underline{q}_t) \max_{A_t} V(A_t | \underline{q}'_t) \\ &= \sum_{z_t} \bar{P}(z_t | \underline{q}_t) \max_{a_t} \left\{ R(a_t, x_t) + \sum_{x'_{t+1}} \bar{P}(x_{t+1} | x_t, a_t, \underline{q}'_t) V[x_{t+1}, \underline{q}_{t+1}] \right\}. \end{aligned}$$

Supporting information

S1 Fig. Trajectories for annual and biennial monitoring for two different models. Model 0 assumes routine and intensive burns are equally effective in setting back succession. Model 1 assume intensive burn is more effective.
(TIF)

S1 File. Transition probabilities of Florida scrub and comparison of annual and biennial monitoring.
(DOCX)

Acknowledgments

We greatly appreciate the assistance of David Breininger, Innovative Health Applications, LCC, for providing information concerning the dynamics of Florida scrub. Any use of trade, firm, or product names is for descriptive purposes only and does not imply endorsement by the U.S. Government.

Author Contributions

Conceptualization: Byron K. Williams, Fred A. Johnson.

Formal analysis: Byron K. Williams, Fred A. Johnson.

Investigation: Byron K. Williams, Fred A. Johnson.

Methodology: Byron K. Williams.

Visualization: Fred A. Johnson.

Writing – original draft: Byron K. Williams, Fred A. Johnson.

Writing – review & editing: Byron K. Williams.

References

1. Williams BK, Johnson FA. Confronting dynamics and uncertainty in optimal decision making for conservation. Environmental Research Letters 2013; 8:025004.
2. Doremus H. Adaptive management as an information problem. North Carolina Law Review 2011; 89:1455–1495.
3. McAllister MK, Pikitch EK. A Bayesian approach to choosing a design for surveying fishery resources: Application to the eastern Bering Sea trawl survey. Canadian Journal of Fisheries and Aquatic Science 1996; 54:301–311.
4. McAllister MK, Kirkwood GP. Bayesian Stock assessment: A review and example application using the logistic model. Journal of Marine Science 1998; 55:1031–1060.
5. Raiffa H, Schlaifer RO. Applied statistical decision theory. Boston, MA: Harvard University Press; 1961.
6. Quirk JP. Intermediate microeconomics. New York: Science Review Associates; 1976.
7. Dakins ME, Toll JE, Small MJ, Brand KP. Risk-based environmental remediation: Bayesian Monte Carlo analysis and the expected value of sample information. Risk Analysis 1996; 16:67–79. PMID: 8868224
8. Yokota F, Thompson KM. Value of information literature analysis: a review of applications in health risk management. Medical Decision Making 2004; 24:287–298. https://doi.org/10.1177/0272989X04263157 PMID: 15155018
9. Yakota F, Thompson KM. Value of Information Analysis in Environmental Health Risk Management Decisions: Past, Present, and Future. Risk Analysis 2004; 24:635–650. https://doi.org/10.1111/j.0272-4332.2004.00464.x PMID: 15209935
10. Canessa S, Guillera-Arroita G, Lahoz-Monfort JJ, Southwell DM, Armstrong DP, Chades I, et al. When do we need more data? A primer on calculating the value of information for applied ecologists. Methods in Ecology and Evolution 2015; 6: 1219–1228.
11. Williams BK, Johnson FA. Uncertainty and the value of information in natural resource management: Technical developments and application to pink-footed geese. Ecology and Evolution 2015; 5:466–474. https://doi.org/10.1002/ece3.1363 PMID: 25691972
12. Keisler JM, Collier ZA, Chu E, Sinatra N, Linkov I. Value of information analysis: the state of application. Environment Systems and Decisions 2014; 34:3–23.

13. Williams BK, Eaton M, Breininger DR. Adaptive resource management and the value of information. Ecological Modelling 2011; 222:3429–3436.
14. Conroy MJ, Barker RJ, Dillingham PW, Fletcher D, Gormley AM, Westbrooke IM. Application of decision theory to conservation management: recovery of Hectors dolphin. Wildlife Research 2008; 35:93–102.
15. Mäntyniemi S, Kuikka S, Rahikainen M, Kell LT, Kaitala V. The value of information in fisheries management: North Sea herring as an example. ICES Journal of Marine Science 2009; 66:2278–2283.
16. Moore JL, Runge MC. Combining structured decision making and value-of-information analyses to identify robust management strategies. Conservation Biology 2012; 26:810–820. https://doi.org/10.1111/j.1523-1739.2012.01907.x PMID: 22862796
17. Johnson FA, Jensen GH, Madsen J, Williams BK. Uncertainty, robustness, and the value of information in managing an expanding Arctic goose population. Ecological Modelling 2014; 273:186–199.
18. Maxwell SL, Rhodes JR, Runge MC, Possingham HP, Ng CF, McDonald-Madden E. How much is new information worth? Evaluating the financial benefit of resolving management uncertainty. Journal of Applied Ecology 2014; 52:12–20.
19. Johnson FA, Smith BJ, Bonneau M, Martin J, Romagosa C, Mazzotti F, et al. Expert Elicitation, Uncertainty, and the Value of Information in Controlling Invasive Species. Ecological Economics 2017; 137:83–90.
20. Runge MC, Converse SJ, Lyons JE. Which uncertainty? Using expert elicitation and expected value of information to design an adaptive program. Biological Conservation 2011; 144:1214–23.
21. Moore AL, Walker L, Runge MC, McDonald-Madden E, McCarthy MA. Two-step adaptive management for choosing between two management actions. Ecological Applications 2017; 27:1210–1222. https://doi.org/10.1002/eap.1515 PMID: 28140503
22. Grantham HS, Moilanen A, Wilson KA, Pressey RL, Rebelo TG, Possingham HP. Diminishing return on investment for biodiversity data in conservation planning. Conservation Letters 2009; 1:190–198.
23. Shea K, Tildesley MJ, Runge MC, Fonnesbeck CF, Ferrari MJ. Adaptive Management and the Value of Information: Learning Via Intervention in Epidemiology. PLOS Biology 2014; 12:1–11.
24. Williams BK, Johnson FA. Value of information and natural resources decision making. Wildlife Society Bulletin 2015; https://doi.org/10.1002/wsb.575
25. Williams BK. Markov decision processes in natural resources management: Observability and uncertainty. Ecological Modelling 2009; 220:830–840.
26. Fackler P. Structural and observational uncertainty in environmental and natural resource management. International Review of Environmental and Resource Economics 2014; 7:109–139.
27. Williams BK, Nichols JD, Conroy MJ. Analysis and Management of Animal Populations. San Diego, CA: Academic Press; 2002.
28. Puterman ML. Markov decision processes: Discrete stochastic dynamic programming. New York, USA: John Wiley and Sons; 1994.
29. Williams BK. Adaptive management of natural resources: Framework and issues. Journal of Environmental Management 2011; 2011:1346–1353.
30. Williams BK, Brown ED. Adaptive management: From more talk to real action. Environmental Management 2014; 53:465–479. https://doi.org/10.1007/s00267-013-0205-7 PMID: 24271618
31. Nichols JD, Williams BK. Adaptive management. In: El-Shahaarwi AH, Piegorsch W, editors. Encyclopedia of Environmetrics. New York: John Wiley and Sons; 2012.
32. Lee PM. Bayesian Statistics: An Introduction. London, UK: Edward Arnold Publishers; 1989.
33. Williams BK. Integrating external and internal learning in resource management. Journal of Wildlife Management 2015; 79:148–155.
34. Walters CJ. Adaptive management of renewable resources. Caldwell, NJ: Blackburn Press; 1986.
35. Williams BK. Passive and active adaptive management: Approaches and an example. Journal of Environmental Management 2011; 92:1371–1378. https://doi.org/10.1016/j.jenvman.2010.10.039 PMID: 21074930
36. Berger JO. Statistical Decision Theory and Bayesian Analysis. New York: Springer-Verlag; 1985.
37. Root KV. The effects of habitat quality, connectivity, and catastrophes on a threatened species. Ecological Applications 1998; 8:854–865.
38. Stith BM, Fitzpatrick JW, Woolfenden GE, Pranty B. Classification and conservation of metapopulations: a case study of the Florida scrub-jay. In: McCullough D.R., editor. Metapopulations and wildlife conservation. Washington, DC: Island Press; 1996. Pp. 187–215
39. Foster TE, Schmalzer PA. The effect of season of fire on the recovery of Florida scrub. Proceedings of the 2nd International Wildland Fire Ecology and Fire Management Congress 2003; http://ams.confex.com/ams/FIRE2003/techprogram/paper_65301.htm (accessed 29 December 2017).

40. Breininger DR, Carter. Territory quality transitions and source-sink dynamics in a Florida Scrub-Jay population. Ecological Applications 2003; 13:516–529.

41. Breininger DR, Larson VL, Duncan BA, Smith RB. Linking habitat suitability to demographic success in Florida scrub-jays. Wildlife Society Bulletin 1998; 26:118–128.

42. Breininger DR, Larson VL, Duncan BA, Smith RB, Oddy DM, Goodchild M. Landscape patterns in Florida scrub jay habitat preference and demography. Conservation Biology 1995; 9:1442–1453.

43. Breininger DR, Oddy DM. Do habitat potential, population density, and fires influence Scrub-Jay source-sink dynamics? Ecological Applications 2004; 14:1079–1089.

44. Johnson FA, Williams BK. A decision-analytic approach to adaptive resource management. In Allen CR, Garmestani AS (eds.). Adaptive Management of Social-Ecological Systems. Houten, Netherlands: Springer; 2015.

45. Moore AL, McCarthy MA. On valuing information in adaptive-management models. Conservation Biology 2010; 24:984–993. https://doi.org/10.1111/j.1523-1739.2009.01443.x PMID: 20136870

46. Williams BK, Johnson FA. Frequencies of decision making and monitoring in adaptive resource management. PLoS ONE 2017; 12: e0182934. https://doi.org/10.1371/journal.pone.0182934 PMID: 28800591

47. McDonald AD, Smith ADM. A tutorial on evaluating expected returns from research for fishery management using Bayes' theorem. Natural Resources Modeling 1997; 10:185–215.

48. Schwartz MW, Cook CN, Pressey RL, Pullin AS, Runge MC, Salafsky N, Sutherland WJ, Williamson M. A. Decision support frameworks and tools for conservation. Conservation Letters 2017; https://doi.org/10.1111/conl.12385

49. Johnson FA, Hagan G, Palmer WE, Kemmerer M. Uncertainty, robustness, and the value of information in managing a population of northern bobwhites. Journal of Wildlife Management 2014; 78:531–539.

50. Nichols JD, Johnson FA, Williams BK, Boomer GS. On formally integrating science and policy: walking the walk. Journal of Applied Ecology 2015; 52: 539–543.
